# Does Revision Anterior Cruciate Ligament (ACL) Reconstruction Provide Similar Clinical Outcomes to Primary ACL Reconstruction? A Systematic Review and Meta‐Analysis

**DOI:** 10.1111/os.12638

**Published:** 2020-08-13

**Authors:** Xu Yan, Xiong‐gang Yang, Jiang‐tao Feng, Bin Liu, Yong‐cheng Hu

**Affiliations:** ^1^ Department of Orthopedics Emergency Tianjin Hospital Tianjin China; ^2^ Department of Orthopedic Oncology Tianjin Hospital Tianjin China; ^3^ Center for Medical Device Evaluation NMPA Beijing China

**Keywords:** Anterior cruciate ligament, Clinical outcome, Primary reconstruction, Revision reconstruction

## Abstract

More revisionary reconstruction procedures are required following failing anterior cruciate ligament (ACL) reconstructions, which are often regarded as a technique challenge with very limited goals. This study will be performed to compare the outcomes between groups of primary and revision knee reconstruction. Two observers conducted the literature retrieval from the platforms of PubMed, Embase, and CENTRAL. Studies which compared knee function and stability between primary and revisionary reconstructions were included. The data was synthesized by meta‐analysis with fixed‐ or random‐effects models as appropriate. A total of 10 eligible studies were included with 954 subjects in the primary group and 378 in the revision group. The International Knee Documentation Committee International Knee Documentation Committee (IKDC) subscores, side‐to‐side difference, and Lysholm score were demonstrated to be significantly improved at final follow‐up in both groups, while Tegner score was not. The overall IKDC, Knee injury and Osteoarthritis Outcome Score (KOOS), and Lysholm scores were significantly inferior in the revision group compared to the primary group. However, knee laxity according to side‐to‐side difference was demonstrated to be similar between the two groups. Revision ACL reconstruction (RACLR) could provide patients with excellent restoration of knee outcomes compared to the status before revision. Also, while knee function in the revision group was inferior to the primary group, knee stability was equivalent between the two groups at the final follow‐up.

## Introduction

Reconstruction of anterior cruciate ligament (ACL) has become a very common procedure in orthopaedic surgery.^1^ And when suffering from grafts which have failed, causing issues such as recurrent symptomatic laxity, arthritis and pain problems, loss of motion and extensor mechanism dysfunction after primary ACLR (PACLR), a revision procedure is required.^2^ It has been reported that there is a total failure rate of 10%‐15% for PACLR at short‐term follow‐up, while long‐term failure has been reported to be as high as 27%.^3^, ^4^ In Australia, former literature has presented that the annual incidence of PACLR has been increased by 43% (from 54.0 to 77.4 per 100 000 population per year), and by 74% among those under 25 years of age (from 52.6 to 91.4 per 100 000 population per year), during the past 15 years. Meanwhile, the annual incidence of revision ACL reconstruction (RACLR) has also been increased from 2.49 to 5.65 per 100 000 population.^5^

RACLR surgery is often regarded as a technical challenge and is considered to be a salvage procedure with very limited goals.^6^, ^7^, ^8^ There are several technical problems during revision procedure, such as graft selection, graft replacement and fixation, and single‐ or two‐stage reconstruction.^9^, ^10^, ^11^ In revision surgery, bone tunnels are inevitably enlarged after the removal of primary grafts, particularly when the position of formerly placed tunnels completely or incompletely overlap with the correct anatomic footprint of the ACL.^12^ It is generally accepted that enlarged bone tunnels with a diameter of more than 15 mm and 10–15 mm with an irregular shape secondary to osteolysis during RACLR would require bone grafting in a one‐ or two‐stage procedure.^13^, ^14^, ^15^, ^16^ Like the primary reconstruction, revision procedures should select a suitable type of graft and place the ligament graft in an anatomical position with a stable fixation. Though the revision surgery is accompanied by a lot of technical troubles, recent research related to RACLR has proposed that satisfactory and favorable clinical outcomes, which are comparable to that of PACLR procedures, can also be obtained, as the techniques and options for suitable ACLR continue to improve.^17^, ^18^ However, only a few studies have focused on the outcome comparison between the primary and revisionary ACL reconstruction groups and, in these studies, a small number of patients were involved for analyzing. Thus, the exact knee outcomes are not yet very clear for the revision procedures of ACL reconstruction when compared to the primary procedures.

In this study, we would like to observe the patientsʼ expectancy of RACLR at final follow‐up vs pre‐operation, and compare the knee function and stability evaluations between groups of PACLR and RACLR through a performed systematic review and meta‐analysis.

## Methods

### *Data Sources and Study Searches*

This review was conducted according to the guidelines outlined in the Preferred Reporting Items for Systematic Reviews and Meta‐analysis (PRISMA) statement. Two individual researchers conducted the platform searches for potential eligible research on the PubMed, Embase, and the Cochrane Central Register of Controlled Trials (CENTRAL) databases from the inception dates to 12 May 2018. Literature retrieval was carried out through a combined search using subject terms (“MeSH” on PubMed and CENTRAL, and “Emtree” on Embase), free terms, and the following keywords: “Primary reconstruction,” “Revision,” and “Anterior cruciate ligament reconstruction”. The searching strategies performed were presented in Appendix S1. Additionally, some other reference studies of relative articles and reviews were screened and hand‐searched for possible inclusion.

### *Inclusion and Exclusion Criteria*

Studies were selected based on the following inclusion criteria: (i) studies comparing clinical results between patients treated with RACLR and patients involved with revision procedures after PACLR; and (ii) studies designed as observational or interventional research, including case‐control study, cross‐sectional study, and clinical‐controlled study. Exclusion criteria: (i) duplicated studies; and (ii) studies designed as literature review, systematic review, and/ or meta‐analysis, case‐series or case report, letter to editors, and conference abstract.

### *Study Selection*

After merging duplicated studies, two researchers independently reviewed the titles/abstracts and full texts of studies, successively. The whole process of study selection was strictly in accordance with the inclusion and exclusion criteria, and all the disagreements were discussed by the two review authors, who reached a consensus. When necessary, the third senior researcher would take part into the resolving of disagreements.

### *Data Extraction and Quality Assessment of Included Studies*

Two authors independently extracted the following information from each included study: (i) study characteristics: lead author, publication year, study design, lead authorʼs country, study period, and follow‐up; (ii) patients information: number of patients, male percentage, age at operation and meniscal injury, and cartilage status at operation; (iii) operation information: graft selection, reconstruction and fixation technique, and revision stage; (iv) status of knee function and stability before operation and at final follow‐up: International Knee Documentation Committee (IKDC) evaluation, Knee injury and Osteoarthritis Outcome Score (KOOS), side‐to‐side difference by KT‐1000/ KT‐2000, Lysholm score, and Tegner score (in the evaluation by IKDC, data referring to the objective IKDC score, Lachman test, manual anterior drawer test, and pivot shift test were extracted; KOOS, which contains a total of five compartments including pain, symptom, ability of daily life, sport, and quality of life, was extracted in detail; and the side‐to‐side differential laxity measured through various types of arthrometers such as KT‐1000, KT‐2000 or GNRB was recorded.). We figured out cause of diversity on obtained information and resolved disagreement after discussion. The process of data extraction was conducted according to the checklists of data collectio proposed by the Cochrane Collaboration.

The Newcastle‐Ottawa Scale (NOS) was used for assessment on methodological quality and risk of bias of case‐control studies and cohort studies.^19^ This scale employs a nine‐stars system that assesses three domains: patient selection, comparability of study groups, and ascertainment of study outcome. The quality assessment checklist proposed by the Agency for Healthcare Research and Quality (AHRQ) was used to assess the quality of cross‐section studies, which consisted of a total of 11 items.^20^

### *Statistical Analysis*

The data referring to evaluations through IKDC, KOOS, and other scores were compared between groups of PACLR and RACLR and between values at pre‐operation and final follow‐up. Chi‐square test and non‐parametric Wilcoxon rank‐sum test were conducted for categorical counting data and ordered categorical data, respectively. Exploratory meta‐analyses were performed using mean difference (MD) as effect size. In cases of studies presenting the median and range value, the calculations spreadsheet was used to assist us in estimating the mean and SD value according to Hozo.^21^ The heterogeneity was tested with I^2^, and, in cases with significant heterogeneity (I^2^ > 50%), random‐effect model and sensitivity analysis were employed, while fixed‐effect model was selected when presenting with excellent homogeneity.^22^ Funnel plot was used to detect the existing publication bias.^23^ The statistical significance was defined at a two‐sided *P*‐value of less than 0.05. The statistical procedures were conducted through the software SPSS version 23.0 (SPSS Inc., Chicago, IL, USA) and Revman version 5.3 (Cochrane Collaboration).

## Results

### *Study Retrieving and Selection*

**Fig 1:**
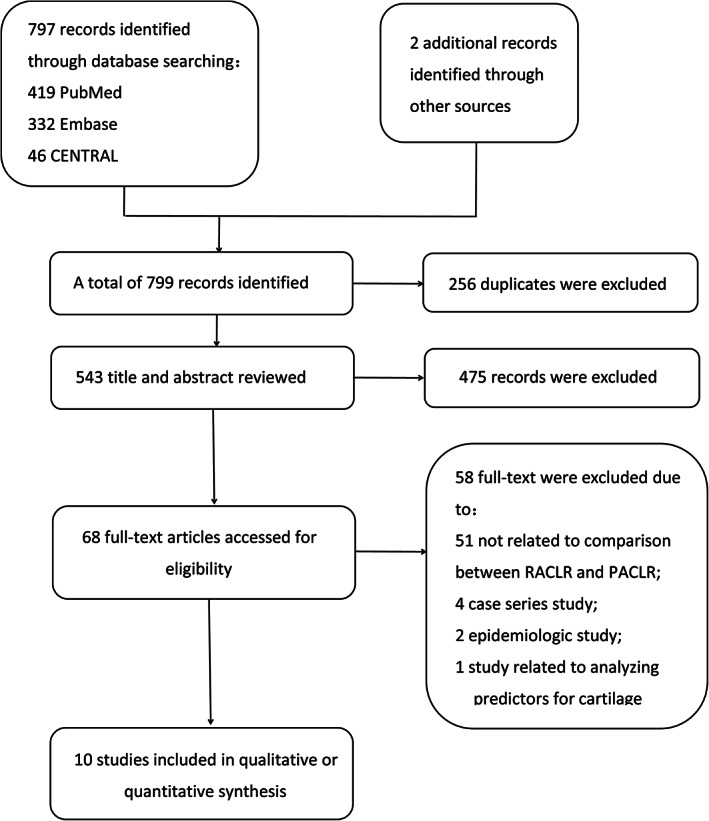
Flowchart describing the literature search and study selection.

A flowchart of identification and the selection of eligible studies was presented in Fig. 1. The primary retrieving on the platforms identified a total of 797 potentially eligible records. In addition, another two studies were screened and hand‐searched for possible inclusion. A total of 256 duplicates were excluded and then titles and abstracts of 543 records were screened for inclusion. Only 68 full texts remained for final selection. Finally, 10 studies^9^, ^13^, ^18^, ^24^, ^25^, ^26^, ^27^, ^28^, ^29^, ^30^ were included for eligibility, while the other 58 full texts were excluded.

### *General Information of Included Studies and Patients*

A summary of included studies is shown in Table 1. All of the studies were demonstrated to be with a favorable quality by NOS (average: 7.6 ± 0.9) or AHRQ checklist (a score of 10 in the cross‐sectional study^24^). All studies were followed with a mean or median period of more than 2 years, and three^13^, ^24^, ^25^ of them were followed for more than 5 years. A total of 954 subjects in PACLR group and 378 in RACLR group were enrolled, with male percentages of 64.3% and 65.3% in two groups, respectively. Meniscal injury and cartilage status were reported in seven studies.^9^, ^13^, ^24^, ^26^, ^27^, ^28^, ^29^ And there were 347 (43.8%) medial and 264 (33.3%) lateral meniscal injuries recorded in the PACLR group among 793 patients, while 137 (60.4%) medial and 79 (34.8%) lateral meniscal injuries in RACLR group among 227 patients. A significantly higher incidence of medial meniscal injury was presented in the PACLR group than the RACLR group (χ^2^ = 19.49, *P* < 0.001) while a non‐significant difference was presented on the lateral meniscal injury (χ^2^ = 0.18, *P* = 0.671). In two of the studies,^9^, ^28^ 127 (20.7%) and 35 (31.5%) patients with cartilage damage were recorded in PACLR and RACLR groups, respectively. A significantly higher incidence of cartilage damage was presented in the RACLR group (χ^2^ = 6.375, *P* = 0.012).

**TABLE 1:** Summary of studies and patients

Author	Study design	Country	Study period	Type of ACLR	Patients (N)	Dropped (N)	Male %	Age	Meniscal injury	Cartilage status	Quality of studies
Kievit AJ, 2013^24^	Cross‐sectional study	Netherlands	1997–2009	PACLR	27	3	59.3	median:33.1(19–57)	Total: 59.0%	median:0 (0–3)†	10‡
				RACLR	25	5	72.0	median:39.9(20–55)	Total:88.0%	median:2 (0–3)†	
Ahn JH, 2008^9^	Case control study	Korea	1997–2005	PACLR	117	0	N/A	mean: 29.1(15–54)	M:61.5%; L:35.9%	Cartilage damage:20.5%	9
				RACLR	56	3	78.2	mean: 31.6(21–55)	M:48.2%; L:25.0%	Cartilage damage:21.4%	
Gifstad T, 2012^25^	Case control study	Norway	1993–2003	PACLR	52	4	43.0	mean: 36(20–57)	N/A	N/A	7
				RACLR	56	13	44.0	mean: 34(20–56)	N/A	N/A	
Kartus J, 1998^26^	Case control study	USA	N/A	PACLR	12	0	41.7	median: 27(19–32)	N/A	N/A	8
				RACLR	12	0	41.7	median: 27(23–33)	L + M:50.0%; M:41.7%	Mild degeneration: 58.3%	
				RACLR	12	0	41.7	median: 27(24–33)	L + M:16.7%; M:41.7%; L:33.3%	Mild degeneration: 50.0%	
Tomihara T, 2017^27^	Case control study	Japan	2007–2013	PACLR	44	0	68.2	mean: 23.2(16–39)	M:52.3%;L:22.7%; Total:61.4%	Grade 2 or higher: 47.7%†	8
				RACLR	22	0	68.2	mean: 22.3(16–39)	M:81.8%;L:27.3%; Total:81.8%	Grade 2 or higher: 86.4%†	
Weiler A, 2007^18^	Case control study	Germany	1997–2005	PACLR	50	0	62.0	mean: 30 ± 8	N/A	N/A	7
				RACLR	50	17	62.0	mean: 31 ± 8	N/A	N/A	
Lefevre N, 2016^28^	prospective cohort study	France	2012–2014	PACLR	497	N/A	67.0	mean:30.1 ± 8.4	M:33.0%; L:32.0%	Cartilage damage: 20.7%	8
				RACLR	55	N/A	72.7	mean:31.6 ± 8.4	M:56.4%; L:36.4%	Cartilage damage: 41.8%	
Muneta T, 2010^29^	Case control study	Japan	1995–2006	PACLR	86	19	58.1	mean:24(14–46)	M:41.9%;L:33.3%	full‐thickness injuries:1.9%	7
				RACLR	21	0	66.7	mean:27(16–40)	M:57.1%; L:33.3%	full‐thickness injuries:9.5%	
Thomas NP, 2005^13^	Case control study	UK	1993–2000	PACLR	49	0	75.0	mean:32.9	M:51.0%; L:24.5%	PFJ:46.9%; M:44.9%; L:26.5%	8
				RACLR	49	0	75.5	mean:32.9	M:85.7%; L:53.1%	PFJ:91.8%; M:61.2%; L:91.8%	
Niki Y, 2010^30^	Case control study	Japan	2005–2007	PACLR	20	0	70.0	mean:28 ± 7(19–46)	N/A	N/A	6
				RACLR	20	0	70.0	mean:29 ± 8(14–45)	N/A	N/A	

Abbreviations: L, lateral; M, medial; PACLR, primary ACL reconstruction; PFJ, patellofemoral joint; RACLR, revision ACL reconstruction

† The cartilage status was graded according to the International Cartilage Repair Society grading scale.

‡ Quality of the study was assessed with the quality assessment checklist proposed by the Agency for Healthcare Research and Quality (AHRQ), while all of the other studies were assessed by the Newcastle‐Ottawa Scale (NOS).

### *Summary of Operation*

**Fig 2:**
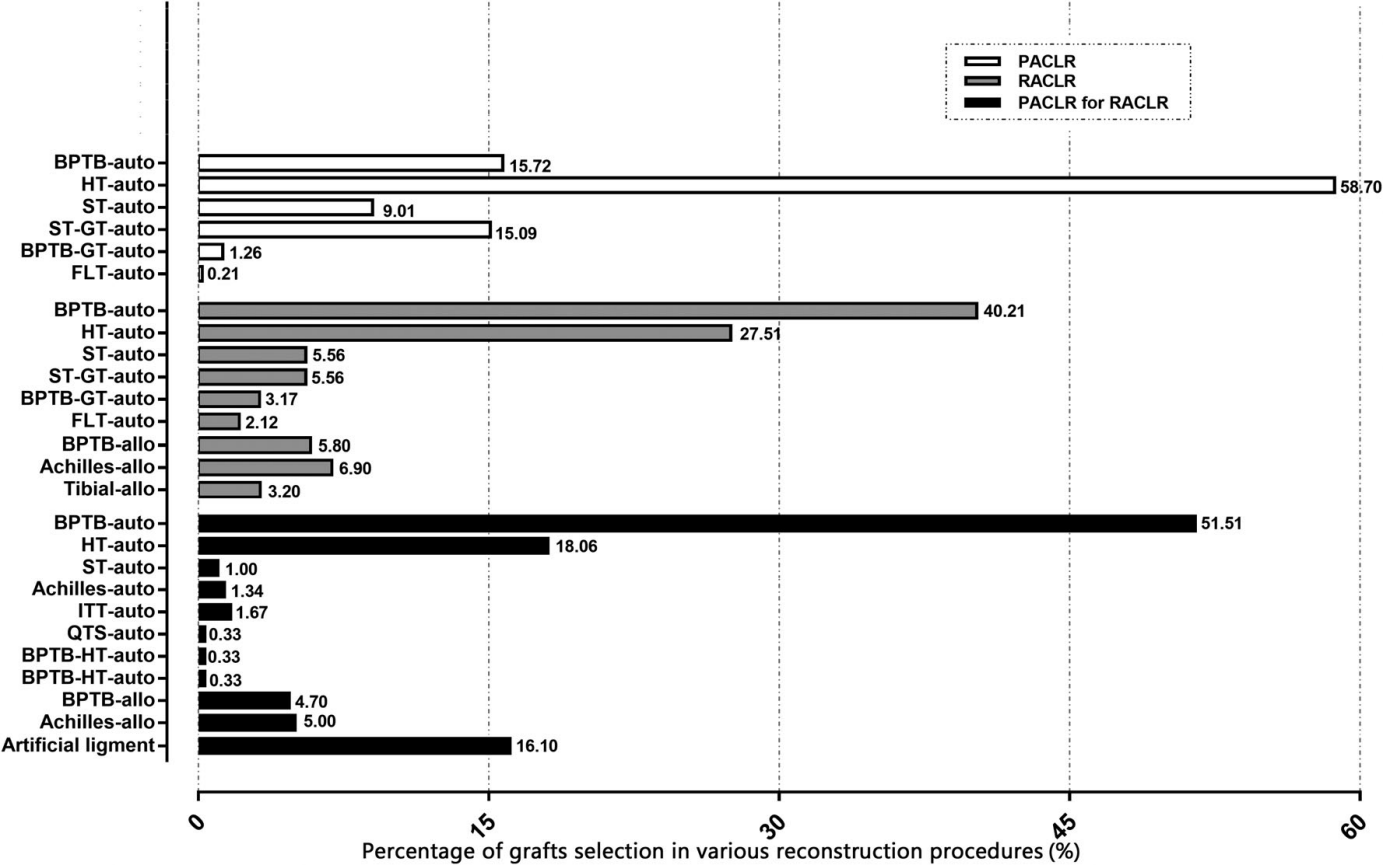
The percentage of each type of grafts selected for various reconstruction procedures. BPTB, bone‐patellar tendon‐bone; FLT, tensor fasciae latae tendon; GT, gracilis tendon; HT, hamstring tendon; ITT, iliotibial tract; PACLR, primary ACL reconstruction; RACLR, revision ACL reconstruction; auto, autograft; allo, allograft; ST, semitendinosus tendon; QTS, quadriceps tendon substitute.

A summary of operations were presented in Table 2. A total of 271 (83.9%) single‐stage and 52 (16.1%) double‐stage revision operations were performed in the RACLR group, respectively. The grafts selection in PACLR, RACLR, and primary reconstruction in the RACLR group was presented in Fig. 2. All of the patients were reconstructed with autograft in the primary reconstruction group. In the revision reconstruction group, 84.1% of the patients were treated with autograft and the rest (15.9%) with allograft. Regarding the RACLR group, autograft, allograft, and artificial ligament were respectively applied to the primary procedure in 74.57%, 9.7%, and 16.1% of the patients.

**TABLE 2:** Summary of operations

Author	Type of ACLR	Patients (N)	Graft Selection	Graft used in PACLR for RACLR group	Technique of reconstruction	Fixation method	Revision stage
Kievit AJ, 2013^24^	PACLR	27	Auto: ST‐GT:27	N/A	Transtibial single‐bundle	F: EndoButton; T: staples	1‐stage
	RACLR	25	Allo: Tibial: 12;Achilles:11; BPTB:2		Transtibial single‐bundle		
Ahn JH, 2008^9^	PACLR	117	Auto: ST‐GT:117	Auto:BPTB:13, HT:5, Achilles:4; Allo:Achilles:15, BPTB:14; Artificial:5	Arthroscopic transtibial double‐looped	F: 2 bioabsorbable cross‐pins; T: bio‐interference screw +post tie	54 1‐stage; 2 2‐stage
	RACLR	56	Auto: ST‐GT:21; Allo: BPTB:20, Achilles:15		Arthroscopic transtibial double‐looped		
Gifstad T, 2012^25^	PACLR	52	Auto: BPTB:44, HT:8	Auto:BPTB:54;HT:2	Transtibial double‐looped	N/A	55 1‐stage; 1 2‐stage
	RACLR	56	Auto: BPTB:54, HT:2		Transtibial single‐bundle		
Kartus J, 1998^26^	PACLR	12	Auto: Ipsilateral PT:12	Ipsilateral PT Auto:24	Arthroscopic reconstruction	F & T: interference screw	1‐stage
	RACLR	12	Auto: Ipsilateral PT:12		Arthroscopic reconstruction		
	RACLR	12	Auto: Contralateral PT:12		Arthroscopic reconstruction		
Tomihara T, 2017^27^	PACLR	44	Auto: BPTB:44	HT Auto:22	Transtibial double‐bundle	F:Endobutton CLs; T: 2 Spike Plates	1‐stage
	RACLR	22	Auto: BPTB:22		Transtibial double‐bundle		
Weiler A, 2007^18^	PACLR	50	Auto: HT:50	Auto:BPTB:30, HT:19; Synthetic:1	Arthroscopic quadrupled tendons	F&T: hybrid (bioabsorbable interference screw, EndoPearl device and suture)	1‐stage
	RACLR	50	Auto: HT:50		Arthroscopic quadrupled tendons		
Lefevre N, 2016^28^	PACLR	497	Auto: BPTB:27, HT:468, FLT:2	N/A	Arthroscopic single‐bundle	F: nonabsorbable screw /Endobutton /interference screw; T: resorbable screw	N/A
	RACLR	55	Auto: BPTB:29, HT:18, FLT:8		Arthroscopic single‐bundle		
Muneta T, 2010^29^	PACLR	86	Auto: ST:86	Auto:BPTB:3, HT:2, ITT:5, QTS:1, ST:3, BPTB+HT:1; Synthetic:2; Allo+synthetic:1; ITT + synthetic:4	Arthroscopic doulble‐bundle	F& T: EndoButton	1‐stage
	RACLR	21	Auto: ST:21		Arthroscopic doulble‐bundle		
Thomas NP, 2005^13^	PACLR	49	Auto: BPTB:15, HT:34	Auto: BPTB:30, HT:4; Synthetic:15	Arthroscopic doulble‐bundle	F:interference screw /Corin anchor /Rigidfix system; T: interference screw /intrafix	2‐stage
	RACLR	49	Auto: BPTB:15, HT:34		Arthroscopic doulble‐bundle		
Niki Y, 2010^30^	PACLR	20	Auto: BPTB‐GT:12, BPTB:8	Synthetic:20	Transtibial single‐ /double‐bundle	F:EndoButton CL BTB device /interference screw; T: N/A	1‐stage
	RACLR	20	Auto: BPTB‐GT:12, BPTB:8		Transtibial single‐ /double‐bundle		

Abbreviations: BPTB, bone‐patellar tendon‐bone; BPTB‐GT, bone‐patellar tendon‐bone+gracilis tendon; F, femoral tunnel; FLT, tensor fasciae latae tendon; HT, hamstring tendon; ITT, iliotibial tract; PT, patellar tendon; ST, semitendinosus tendon; ST‐GT, semitendinosus‐gracilis tendon; T, tibial tunnel; QTS, quadriceps tendon substitute.

### *Knee Outcomes Compared with Pre‐Operative Status*

**Fig 3:**
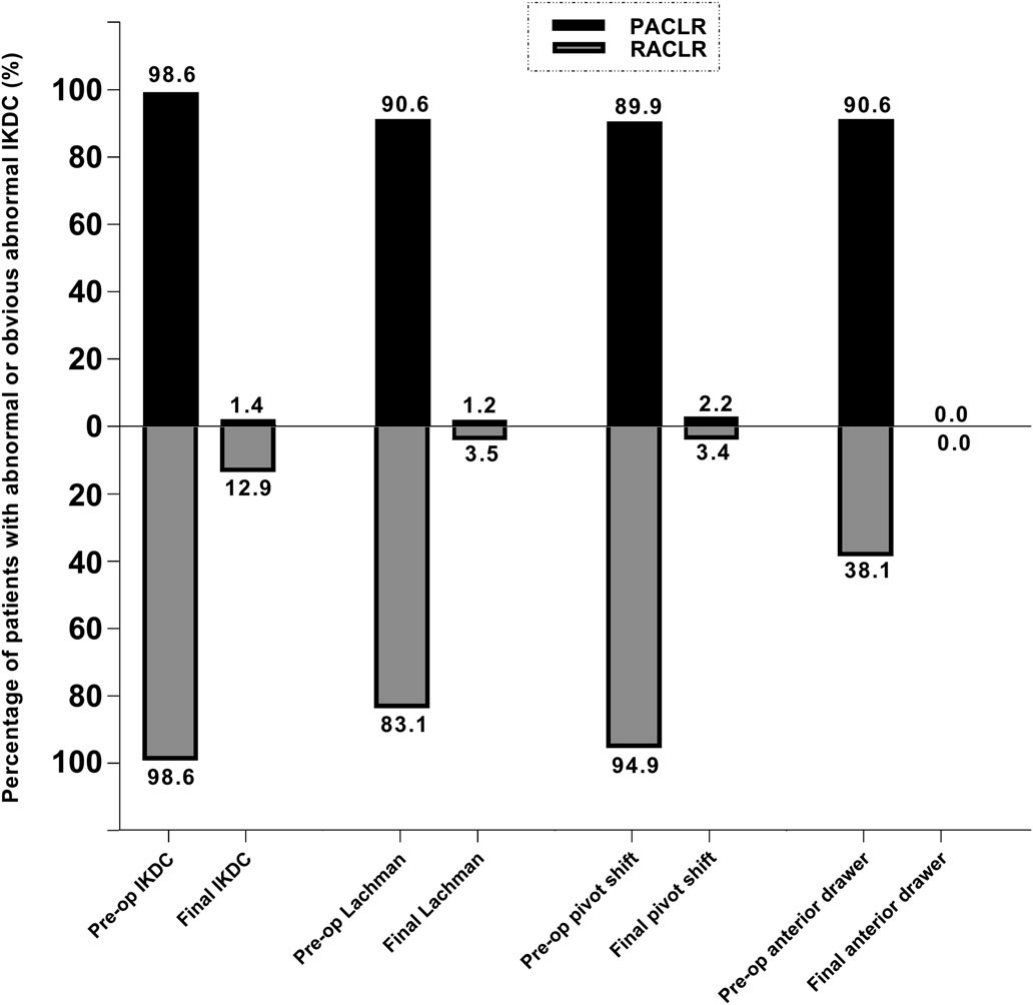
The final knee outcomes by objective International Knee Documentation Committee (IKDC), Lachman test, pivot shift test and anterior drawer teat compared with pre‐operative status for primary and revision ACL reconstruction (RACLR) groups. The height of columns representing the percentages of patients with abnormal or obviously abnormal IKDC subscores. All of the IKDC subscores were obviously improved from pre‐operative status both in primary and revision reconstruction groups.

**Fig 4:**
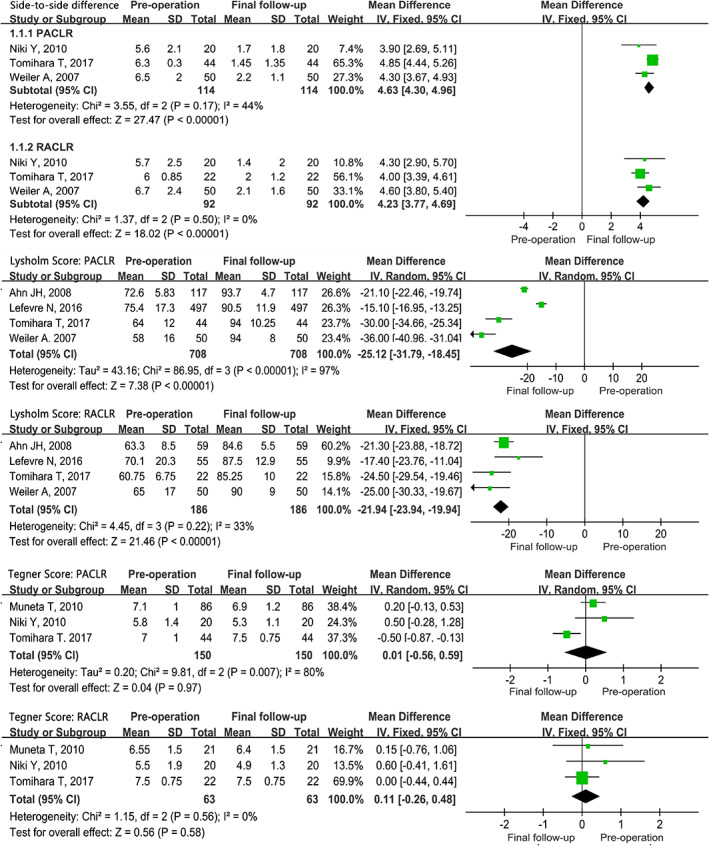
Forest plot of the knee outcomes by side‐to‐side difference, Lysholm score and Tegner score compared with pre‐operative status.

The final knee outcomes by IKDC compared with pre‐operative status were presented in Table 3 and Fig. 3. All of the subscores were obviously improved from the pre‐operative status. At final follow‐up, the patients with abnormality or obvious abnormality on the objective IKDC score, Lachman score, pivot shift test, and anterior drawer test had decreased by 98.6%, 98.7%, 97.5%, and 100% in the PACLR group, and by 87.0%, 96.9%, 96.1%, and 100% in the RACLR group. respectively, when compared to the pre‐operative status. In general, an excellent improvement was recorded in the subscores of IKDC in both the primary and revision groups. The knee outcomes by side‐to‐side difference, Lysholm score, and Tegner score compared with pre‐operative status was presented with forest plots in Fig. 4. In the PACLR group, the MDs on the side‐to‐side difference, Lysholm score, and Tegner score were presented to be −4.63 (CI 95%,‐4.96~−4.30), 25.12 (CI 95%, 18.45~31.79), and −0.01 (CI 95%, −0.59~0.56). And in the RACLR group, the MDs on the side‐to‐side difference, Lysholm score, and Tegner score were presented to be −4.23 (CI 95%, −4.69~−3.77), 21.94 (CI 95%, 19.94~23.94), and −0.11 (CI95%, −0.48~0.26), respectively. The side‐to‐side difference and Lysholm score were significantly improved at final follow‐up while the Tegner score was not improved in both groups. In addition, the MDs on side‐to‐side difference and Lysholm score were similar to each other between the two groups.

**TABLE 3:** International Knee Documentation Committee (IKDC) score (pre‐operation *vs* final follow‐up)

IKDC subscore	PACLR group			RACLR group		
	Pre‐operation‐N(%)	Final follow‐up ‐N(%)	*P* value‡	Pre‐operation‐N(%)	Final follow‐up ‐N (%)	*P* value‡
**Objective IKDC score‐N(%)** ^18^, ^30^						
A/B	1(1.4)	69(98.6)	<0.001	1(1.4)	61(87.1)	<0.001
C/D†	69(98.6)	1(1.4)		69(98.6)	9(12.9)	
**Lachman test (mm)‐N(%)** ^9^, ^29^						
‐1~2/3~5	8(9.4)	85(98.8)	<0.001	13(16.9)	55(96.5)	<0.001
6~10/>10^a^	77(90.6)	1(1.2)		64(83.1)	2(3.5)	
**Pivot shift test‐N(%)** ^9^, ^13^, ^27^, ^29^						
−/+	18(10.1)	175(97.8)	<0.001	7(5.1)	140(96.6)	<0.001
2+/3 +†	160(89.9)	4(2.2)		129(94.9)	5(3.4)	
**Anterior drawer test (mm)‐N(%)** ^29^						
‐1~2/3~5	8(9.4)	86(100)	<0.001	13(61.9)	18(100)	<0.001
6~10/>10^a^	77(90.6)	0(0)		8(38.1)	0(0)	

† According to the IKDC evaluation, objective IKDC score A/B, Lachman test −1~2/3~5 mm, pivot shift test −/+ and anterior drawer test −1~2/3~5 mm were considered to normal or near‐normal, while objective IKDC score C/D, Lachman test 6~10/>10 mm, pivot shift test 2+/3+ and anterior drawer test 6~10/>10 mm were abnormal or obviously abnormal.

‡ Chi‐square test was performed to compare the differences between subscores prior to operation and at final follow‐up, and all of the subscores were demonstrated to be significantly improved at final follow‐up both in PACLR and RACLR group

### *Knee Outcomes of PACLR Group Compared with RACLR Group*

The evaluation of IKDC score at final follow‐up in PACLR and RACLR groups was presented in Table 4. Significant inferior clinical outcomes were recorded in RACLR group regarding objective IKDC score, Lachman test, pivot shift test, and anterior drawer test when compared to PACLR group (*P* < 0.05). At final follow‐up, 6%, 13%, 2%, and 5% of patients presented with an abnormal/obvious abnormal status on the IKDC score, Lachman test, pivot shift test, and anterior drawer test in the PACLR group, respectively, while 22%, 19%, 6%, and 16% presented similarly in RACLR group.

**TABLE 4:** International Knee Documentation Committee (IKDC) score at final follow‐up (revision ACL reconstruction [RACLR] *vs* primary ACL reconstruction [PACLR])

Author	ACLR Type	Patients (n)	Objective IKDC score‐N(%)				Lachman test (mm)‐N(%)				Pivot shift test‐N(%)				Anterior drawer test (mm)‐N(%)			
			A	B	C	D	−1~2	3~5	6~10	>10	‐	1+	2+	3+	−1~2	3~5	6~10	>10
Kievit AJ, 2013^24^	PACLR	27	A + B: 18(67)		C + D: 9(33)		2(7)	19(70)	6(22)	0(0)	20(73)	6(23)	0(0)	1(4)	1(4)	20(74)	6(22)	0(0)
	RACLR	25	A + B: 17(68)		C + D: 8(32)		2(8)	15(60)	7(28)	1(4)	8(32)	13(52)	4(16)	0(0)	4(16)	14(56)	6(24)	1(4)
Ahn JH, 2008^9^	PACLR	117	75(64)	36(31)	6(5)	0(0)	N/A				N/A				N/A			
	RACLR	56	13(23)	35(63)	6(11)	2(4)	34(61)	22(39)	0(0)	0(0)	40(71)	16(29)	0(0)	0(0)				
Gifstad T, 2012^25^	PACLR	52	N/A				−1~5:46(88)		6(12)	0(0)	−~1+:50(96)		2 + ~3+:2(4)		N/A			
	RACLR	56					−1~5:48(86)		7(13)	1(2)	−~1+:45(80)		2 + ~3+:11(20)					
Kartus J, 1998^26^	PACLR	12	5(42)	5(42)	2(16)	0(0)	N/A				N/A				N/A			
	RACLR	12	0(0)	3(25)	7(58)	2(17)												
	RACLR	12	0(0)	7(58)	4(33)	1(8)												
Tomihara T, 2017^27^	PACLR	44	N/A				N/A				35(80)	6(14)	2(5)	1(2)	N/A			
	RACLR	22									16(73)	3(14)	2(9)	1(5)				
Weiler A, 2007^18^	PACLR	50	27(54)	23(46)	0(0)	0(0)	31(62)	19(38)	10(20)	0(0)	41(82)	9(18)	0(0)	0(0)	N/A			
	RACLR	50	21(42)	23(46)	5(10)	1(2)	27(54)	22(44)	1(2)	0(0)	36(72)	12(24)	2(4)	0(0)				
Muneta T, 2010^29^	PACLR	86	N/A				76(88)	9(10)	1(1)	0(0)	77(90)	9(10)	0(0)	0(0)	78(91)	8(9)	0(0)	0(0)
	RACLR	18					15(83)	1(6)	2(11)	0(0)	14(78)	3(17)	1(6)	0(0)	15(83)	3(17)	0(0)	0(0)
Thomas NP, 2005^13^	PACLR	49	24(49)	20(41)	4(8)	1(2)	N/A				44(90)	4((8)	1((2)	0(0)	N/A			
	RACLR	49	12(24)	28(57)	8(16)	1(2)					43(88)	5(10)	1(2)	0(0)				
Niki Y, 2010^30^	PACLR	20	11(55)	8(40)	1(5)	0(0)	N/A				19(95)	1(5)	0(0)	0(0)	N/A			
	RACLR	20	8(40)	6(30)	6(30)	0(0)					17(85)	3(15)	0(0)	0(0)				
**Total**	PACLR	457	142(57)	92(37)	13(5)	1(1)	109(61)	47(26)	23(13)	0(0)	236(86)	35(13)	3(1)	2(1)	79(70)	28(25)	6(5)	0(0)
	RACLR	320	54(27)	102(51)	36(18)	7(4)	44(44)	38(38)	17(17)	2(2)	134(73)	39(21)	10(5)	1((1)	19(44)	17(39)	6(14)	1(2)
**Wilcoxon rank sum test** †			**Z = 7.044, *P* < 0.001**				**Z = 2.737, *P* = 0.006**				**Z = 3.433, *P* = 0.001**				**Z = 3.138, *P* = 0.002**			

† Wilcoxon rank sum test was performed and all of the above IKDC subscores were demonstrated to be significantly inferior in RACLR compared to PACLR group

**Fig 5:**
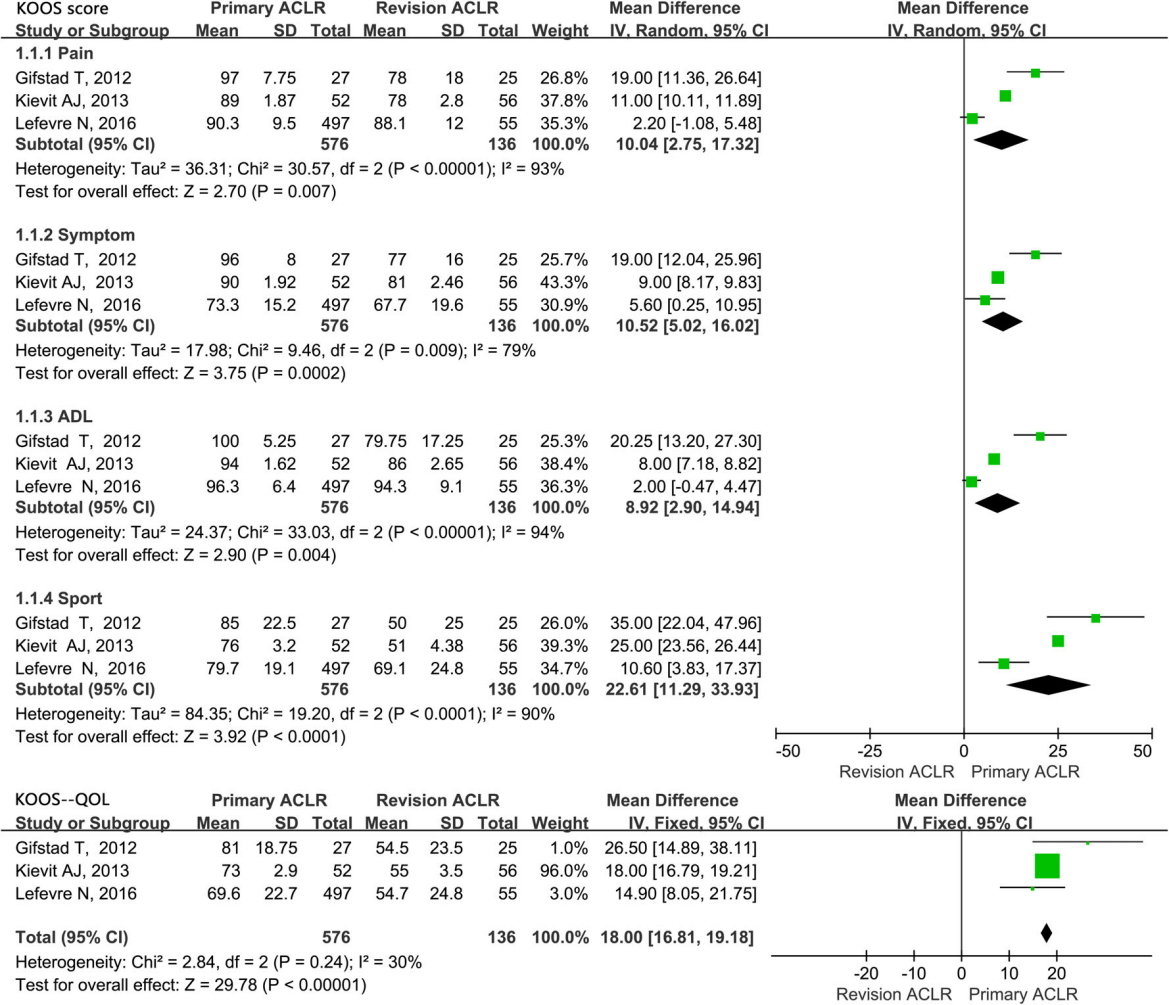
Forest plot of the Knee injury and Osteoarthritis Outcome Score (KOOS) at final follow‐up compared between primary and revision groups.

The evaluation of KOOS at final follow‐up in PACLR and RACLR groups was presented in Fig. 5. Significant higher scores were presented on all of the pain, symptom, ADL, sport, and quality of life subscores in the primary group (*P* < 0.05). The MDs were 10.04 (CI 95%, 2.75~17.32), 10.52 (CI 95%, 5.02~16.02), 8.92 (CI 95%, 2.90~14.94), 22.61 (CI 95%, 11.29~33.93), and 18.00 (CI 95%, 16.81~19.18). respectively.

**Fig 6:**
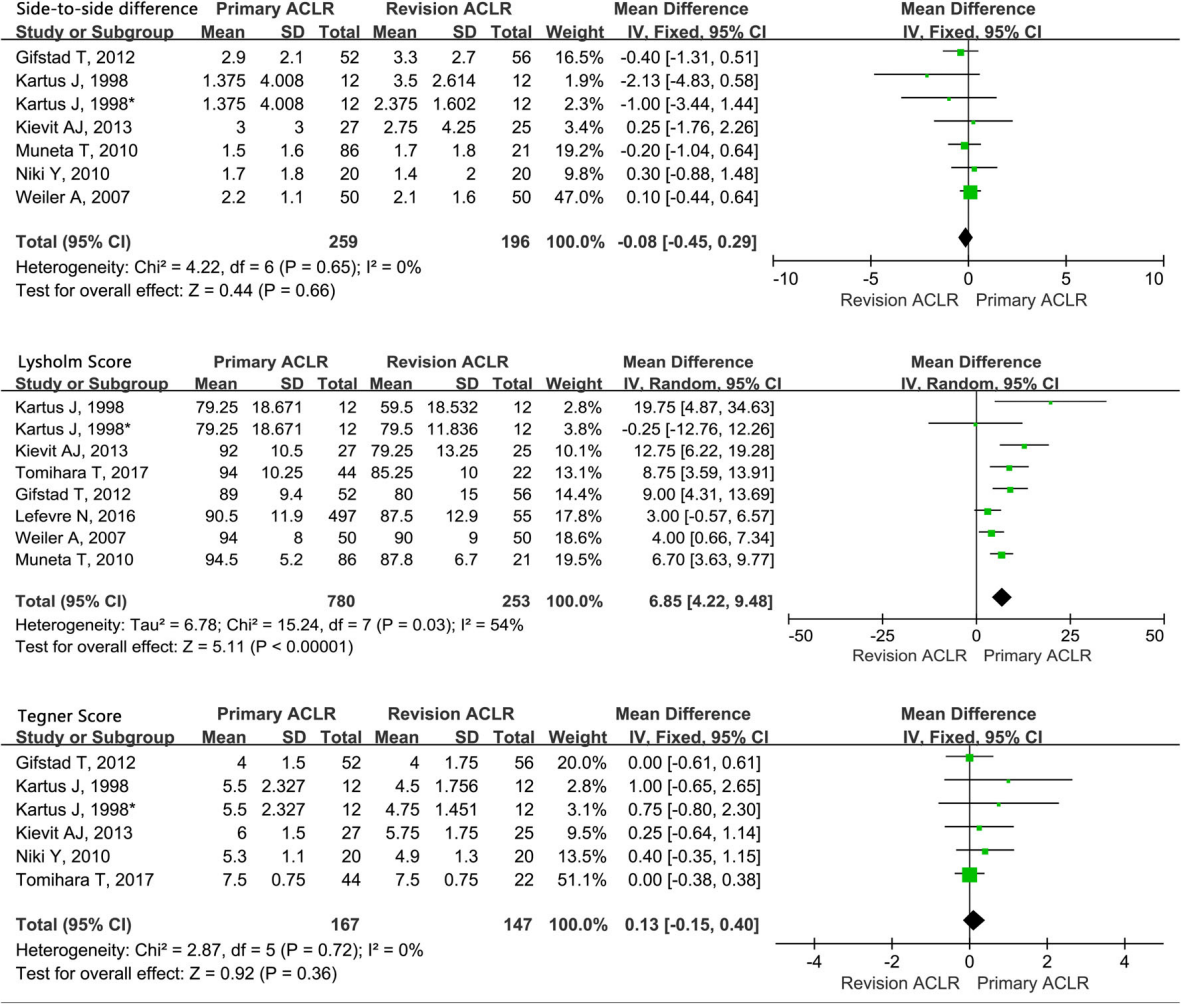
Forest plot of the knee outcomes by side‐to‐side, Lysholm score and Tegner score in revision ACL reconstruction (RACLR) compared with primary ACL reconstruction (PACLR) group.

The knee outcomes by side‐to‐side, Lysholm score, and Tegner score in the RACLR compared with the PACLR group were presented in Fig. 6. A significantly higher Lysholm score was presented in the primary group (MD = 6.85, CI 95%, 3.63~9.77, *P* < 0.001), while the MDs for side‐to‐side difference and Tegner score were non‐significant (*P* > 0.05).

Favorable symmetries were presented in all of the funnel plots which indicated the non‐existence of obvious publication bias.

## Discussion

Revision reconstruction after failure of primary procedure is widely regarded as a technical challenge, especially when bone tunnels are obviously enlarged or irregularly shaped, requiring bone graft in a one‐stage or two‐stage reconstruction.^6^, ^7^, ^8^, ^13^, ^14^, ^15^, ^16^ Thus, the patientʼs expectancy after revision is often adjudged to be unfavorable.^6^, ^7^ However, though many issues are still unavoidable, the recent literature dealing with RACLR has proposed that satisfactory and favorable clinical outcomes can be obtained, which is comparable to PACLR, as the techniques and options for suitable ACLR continue to improve.^17^, ^18^ We know that patient expectations are more likely to be determined by a complex interaction of several biopsychosocial factors.^31^ And a realistic and exact expectation before revision is necessary as it could help surgeons and patients get a clear understanding of the disease situation, which can have a positive influence on patient‐reported outcomes. In the current study, we have identified how much the knee function and stability could be improved after revision reconstruction and whether a compatible clinical outcome could be obtained in the RACLR group when compared to the PACLR group.

As reported in previous literature, revision reconstruction has become an effective treatment option for secondary ACL tears.^12^, ^32^, ^33^, ^34^, ^35^ In the study by Saper,^32^ a good to excellent outcome has been reported in adolescent athletes after revision reconstruction (satisfaction rate, 95.3%; IKDC, 87.5 ± 12.7; Tegner, 7.2 ± 2.0; Lysholm, 93.7 ± 9.8), and 68.4% of the athletes attempting to return to sport returned to their preinjury level of competition. A total of 148 RACLR patients were involved in the study by Diamantopoulos *et al*.,^12^ and significant improvements on the average Lysholm score (88.5 ± 12.4 *vs* 51.5 ± 24.9) and average Tegner activity score (6.3 ± 1.8 *vs* 2.8 ± 1.8) were obtained. At final follow‐up, Grossman *et al*.,^33^ OʼNeill,^34^ and Garofalo *et al*.^35^ reported, in their series, that 86.2%, 84%, and 93%, respectively, of the knees to be normal or near normal by IKDC after revision, which was in accordance with the result in our study (the overall final IKDC qualification showed 87.1% of the knees to be normal or nearly normal in revision group). Thus, in general, revision reconstruction could provide patients with excellent restoration of stability and clinical outcome when compared to the status before revision.

Compared to primary reconstruction, revision reconstruction was often regarded as a less favorable procedure. The inferior outcome of IKDC in the RACLR group compared to the PACLR group has been commonly reported, as well as the outcome of Lysholm score.^9^, ^13^, ^18^, ^30^ The possible reason causing it to be less favorable may be due to the higher rates of meniscal injury and cartilage damage. Wright *et al*.^36^ conducted a cohort study, which enrolled a total of 1205 patients involved with RACLR, to identify the relationship of meniscal and articular cartilage damage to the knee outcomes; this study found that prior lateral meniscectomy and current higher graded changes of the trochlea were associated with worse outcomes at 2 years after revision. In the study by Webster *et al*.,^37^ it was presented that the presence of more severe chondral damage and medial meniscal pathology at the time of RACLR has a negative impact on functional outcomes, activity levels, and return to sport rates. However, there were no differences in any outcome score between patients with and without lateral meniscal pathology. Tomihara *et al*.^27^ also reported a significantly higher incidence of medial meniscus (81.8% *vs* 52.3%) and cartilage injury (86.4% *vs* 47.7%) in the revision group than the primary group, while the difference of lateral meniscal injury (27.3% *vs* 22.7%) between two groups was non‐significant. In our study, a significantly higher incidence of medial meniscal injury (60.4% *vs* 43.8%) and cartilage damage (31.5% *vs* 20.7%) were presented in the PACLR group than the RACLR group, and a non‐significant difference was presented on the lateral meniscal injury (34.8% *vs* 33.3%), which was in accordance with the commonly reported results. It has been described that the normal kinematics of the knee relies upon the integral link between the ACL and the menisci in a former study,^38^ and the importance of the medial meniscus as a secondary stabilizer for anteroposterior translation has been demonstrated by many biomechanical cadaveric studies.^39^, ^40^ Additionally, concurrent medial meniscal injury and cartilage damage are often related to a higher rate of osteoarthritis due to abnormal knee kinematics.^41^, ^42^ Thus, although revision procedures could restore knee outcome and stability to a large extent compared to pre‐operative status, the higher prevalence rate of medial meniscal injury and cartilage damage have caused inferior knee outcomes in the RACLR group when compared to the PACLR group.

However, to our surprise, the pooled MD of side‐to‐side difference was proved to be non‐significant between revision and primary group. In the study of Tomihara *et al*.,^27^ there were no significant differences in KT‐1000 outcomes (2.0 mm *vs* 1.4 mm), pivot shift test, and Tegner score between the two groups. Thus, the author concluded that RACLR provided almost compatible postoperative knee stability with primary ACL reconstruction. In a study by Kievit *et al*.,^24^ no differences were found in anterior drawer, Lachman, or KT‐1000 arthrometer testing between primary and revision groups. Some other studies have also found excellent knee stability according to side‐to‐side difference between the injured and non‐injured sides after revision procedure which was approaching to the primary group.^9^, ^29^ Thus, revision procedures could provide patients in the RACLR group with the equivalent knee stability to those in the PACLR group, as the primary objective of both primary and revision reconstruction is to restore the structural integrity of ACL and stability of the knee joint.

This study, nevertheless, has some limitations. First, primary studies were mainly designed as retrospective case‐control and cross‐sectional studies, not prospective studies of high quality. This may be due to few prospective studies having been carried out on this topic until now. Also, the number of patients enrolled was small, especially in the RACLR group, which may be due to a small number of patients having undergone these revision procedures.

### *Conclusions*

RACLR could provide patients with excellent restoration of the stability and function of the knee when compared to the status before revision. When compared to PACLR, the knee‐function evaluations were inferior in the RACLR group after reconstruction, while knee stability was equivalent between the two groups at final follow‐up.

## Supporting information


**Appendix S1** Searching Strategies performed in retrieving of eligible studies.
