# Supplementary material for: Does Revision Anterior Cruciate Ligament (ACL) Reconstruction Provide Similar Clinical Outcomes to Primary ACL Reconstruction? A Systematic Review and Meta‐Analysis
Source: Orthop Surg. 2020 Aug 13;12(6):1534–46. doi: 10.1111/os.12638 (PMC7767695; doi:10.1111/os.12638)
Supplement: Supplementary file 1 — Appendix S1 Searching Strategies performed in retrieving of eligible studies. [file OS-12-1534-s001.doc]

**Appendix 1. Searching Strategies performed in retrieving of eligible studies**

1. **PubMed**

#1 (Anterior Cruciate Ligament[MeSH]) OR (Anterior Cranial Cruciate Ligament) OR (Cranial Cruciate Ligament) OR (Cranial Cruciate Ligaments) OR (Cruciate Ligament, Cranial) OR (Cruciate Ligaments, Cranial) OR (Ligament, Cranial Cruciate) OR (Ligaments, Cranial Cruciate) OR (Cruciate Ligament, Anterior) OR (Anterior Cruciate Ligaments) OR (Cruciate Ligaments, Anterior) OR (Ligament, Anterior Cruciate) OR (Ligaments, Anterior Cruciate) OR (Anterior Cruciate Ligament Reconstruction)

#2 (primary reconstruction) OR (primary, reconstruction)

#3 (Reoperation[MeSH]) OR (Surgical Revision) OR (Surgery, Repeat) OR (Revision, Surgical) OR (Revision Surgery) OR (Revision Surgeries) OR (Surgery, Revision) OR (Repeat Surgery) OR (Revision, Joint) OR (Joint Revision))

#4 #1 AND #2 AND #3

1. **EMBASE**

#1 ‘Anterior Cruciate Ligament Reconstruction’/exp OR ‘Anterior Cruciate Ligament’ OR ‘Anterior Cranial Cruciate Ligament’ OR ‘Cranial Cruciate Ligament’ OR ‘Cranial Cruciate Ligaments’ OR ‘Cruciate Ligament, Cranial’ OR ‘Cruciate Ligaments, Cranial’ OR ‘Ligament, Cranial Cruciate’ OR ‘Ligaments, Cranial Cruciate’ OR ‘Cruciate Ligament, Anterior’ OR ‘Anterior Cruciate Ligaments’ OR ‘Cruciate Ligaments, Anterior’ OR ‘Ligament, Anterior Cruciate’ OR ‘Ligaments, Anterior Cruciate’ OR ‘ACL’ OR ‘ACLR’

#2 ‘Revision’/exp OR ‘Reoperation’ OR ‘Surgical Revision’ OR ‘Surgery, Repeat’ OR ‘Revision, Surgical’ OR ‘Revision Surgery’ OR ‘Revision Surgeries’ OR ‘Surgery, Revision’ OR ‘Repeat Surgery’ OR ‘Revision, Joint’ OR ‘Joint Revision’

#3 ‘primary reconstruction’ OR ‘primary, reconstruction’ OR ‘primary’

#4 #1 AND #2 AND #3

1. **the Cochrane Central Register of Controlled Trials (CENTRAL)**

#1 MeSH descriptor: [Anterior Cruciate Ligament Reconstruction] explode all trees

#2 Anterior Cruciate Ligament Injury OR Anterior Cruciate Ligament Tear* OR Anterior Cranial Cruciate Ligament OR Cranial Cruciate Ligament* OR Cruciate Ligament*, Cranial OR Ligament*, Cranial Cruciate OR Cruciate Ligament, Anterior OR Anterior Cruciate Ligaments OR Cruciate Ligaments, Anterior OR Ligaments, Anterior Cruciate

#3 #1 OR #2

#4 MeSH descriptor: [Reoperation] explode all trees

#5 Surgical Revision OR Surgery, Repeat OR Revision, Surgical OR Revision Surgery OR Revision Surgeries OR Surgery, Revision OR Repeat Surgery OR Revision, Joint OR Joint Revision

#6 #4 OR #5

#7 primary reconstruction OR primary, reconstruction

#8 #3 and #6 and #7
